# Adjuvant-dependent regulation of interleukin-17 expressing γδ T cells and inhibition of Th2 responses in allergic airways disease

**DOI:** 10.1186/s12931-014-0090-5

**Published:** 2014-08-14

**Authors:** Emily M Nakada, Jichuan Shan, Margaret W Kinyanjui, Elizabeth D Fixman

**Affiliations:** Meakins-Christie Laboratories, McGill University, 3626 St. Urbain Street, Montreal, Quebec H2X 2P2 Canada

**Keywords:** Asthma, Interleukin-17, γδ T cell, Adjuvant, Complete Freund’s adjuvant

## Abstract

**Background:**

Th2 immune responses are linked primarily to mild and moderate asthma, while Th17 cells, Interleukin-17A (IL-17) and neutrophilia have been implicated in more severe forms of disease. How Th2-dependent allergic reactions are influenced by Th17 and IL-17-γδ T cells is poorly understood. In murine models, under some conditions, IL-17 promotes Th2-biased airway inflammatory responses. However, IL-17-γδ T cells have been implicated in the inhibition and resolution of allergic airway inflammation and hyperresponsiveness (AHR).

**Methods:**

We compared airway responses in Balb/c mice sensitized to OVA with (and without) a Th2-skewing aluminum-based adjuvant and the IL-17 skewing, complete Freund’s adjuvant (CFA). AHR was measured invasively by flexiVent, while serum OVA-IgE was quantified by an enzyme immunoassay. Airway inflammatory and cytokine profiles, and cellular sources of IL-17 were assessed from bronchoalveolar lavage and/or lungs. The role of γδ T cells in these responses was addressed in OVA/CFA sensitized mice using a γδ T cell antibody.

**Results:**

Following OVA challenge, all mice exhibited mixed eosinophilic/neutrophilic airway inflammatory profiles and elevated serum OVA-IgE. Whereas OVA/alum sensitized mice had moderate inflammation and AHR, OVA/CFA sensitized mice had significantly greater inflammation but lacked AHR. This correlated with a shift in IL-17 production from CD4^+^ to γδ T cells. Additionally, OVA/CFA sensitized mice, given a γδ TCR stimulatory antibody, showed increased frequencies of IL-17-γδ T cells and diminished airway reactivity and eosinophilia.

**Conclusions:**

Thus, the conditions of antigen sensitization influence the profile of cells that produce IL-17, the balance of which may then modulate the airway inflammatory responses, including AHR. The possibility for IL-17-γδ T cells to reduce AHR and robust eosinophilic inflammation provides evidence that therapeutic approaches focused on stimulating and increasing airway IL-17-γδ T cells may be an effective alternative in treating steroid resistant, severe asthma.

**Electronic supplementary material:**

The online version of this article (doi:10.1186/s12931-014-0090-5) contains supplementary material, which is available to authorized users.

## Background

Asthma is a complex disease characterized by airway inflammation, hyperresponsiveness (AHR) and variable airflow obstruction [1,2] mediated, at least in part, by an aberrant T helper (Th)2 [2-4] and/or Th17 response [5-7]. Th2 cells and the associated cytokines, interleukin (IL)-4, IL-5 and IL-13, are increased in the bronchoalveolar lavage (BAL), sputum and bronchial biopsies of asthmatics and are linked to airway eosinophilia, IgE and reduced lung function [3,4,8,9]. Th17 cells and their prime effector cytokine, IL-17A (hereafter referred to as IL-17), have more recently been implicated in asthma pathogenesis. IL-17 expressing cells are increased in the BAL, sputum, bronchial biopsies and in peripheral blood of asthmatics [5-7] and are correlated with neutrophilic airway inflammation and resistance to corticosteroid treatment in moderate-to-severe asthmatics [10-12]. Although much of the human data is approached from either the Th2 or Th17 perspective, they are unlikely to be mutually exclusive. Asthmatics have been reported to have increases in both Th2- and Th17-related cytokines [13,14] and Cosmi et al. have reported increases in a unique subset of CD4^+^ T cells expressing both IL-4 and IL-17 [15]. Asthmatics can also present with a combined eosinophilic/neutrophilic airway inflammatory profile [16,17], which may reflect a mixed immune response.

Eosinophilic airway inflammation, AHR and mucus production are features of asthma that have been successfully modeled in Th2-driven experimental asthma in the mouse [18,19]. A widely used method of antigen sensitization involves intraperitoneal (IP) injection(s) of ovalbumin (OVA) adsorbed to an aluminum-based adjuvant (hereafter referred to as alum), a potent inducer of the Th2 response [20]. Comparatively, animal studies of IL-17 show an important role for Th17 cells on airway neutrophilia and steroid resistance [21,22]. Intranasal instillations, as well as epicutaneous routes of OVA sensitization, have been shown to induce a more robust IL-17 response compared to IP injections of OVA/alum [23,24]. Furthermore, complete Freund’s adjuvant (CFA), a strong inducer of IL-17 typically used in experimental autoimmune encephalomyelitis models, has seen limited use as an IL-17-skewing adjuvant in murine models of allergic asthma [22,25].

The Th2/IL-17 relationship has been assessed in different Th2-driven models of experimental asthma. A growing body of evidence suggests that IL-17, when sourced from Th17 cells, enhances Th2-induced eosinophilic inflammation and AHR [24,26], whereas we and others have shown that IL-17-γδ T cells negatively regulate these responses [27,28]. We have now compared how two widely used adjuvants, alum and CFA, modulate OVA-induced allergic airways disease. Our primary goals were to induce a mixed Th2/IL-17 inflammatory response and to identify the cellular source(s) of IL-17. Following airway OVA challenge, OVA/alum and OVA/CFA sensitized mice exhibited antigen-induced airway eosinophilia and had similar total IL-17^+^ and Th17 BAL fluid cell numbers. However, the influx of inflammatory cells into the lung, as well as serum OVA-IgE levels, and BAL fluid IL-17-γδ T cells were all significantly enhanced in OVA/CFA over OVA/alum sensitized mice following OVA challenge. Nevertheless, AHR was completely absent in these highly inflamed animals, which corresponded to a shift in IL-17 production from CD4^+^ to γδ T cells. Our secondary goal was to define the role of IL-17-γδ T cells on AHR and inflammation. In mice treated with a γδ TCR stimulatory antibody, the frequency of IL-17-γδ T cells in OVA/CFA sensitized mice was increased. Consistent with a negative regulatory role for these cells, AHR and eosinophilia were both significantly decreased in these mice. Overall, our data demonstrate that the conditions of initial antigen sensitization direct production of IL-17 from different populations of cells, which in turn, may have the ability to modulate Th2-biased airway inflammatory responses. These data also suggest that specific enhancement of IL-17-γδ T cells could inhibit allergic airways responses.

## Methods

### Animals

Wild type Balb/c mice originating from Charles River Laboratories (Montreal, QC, Canada) were bred and used at ages 6–10 weeks at the Meakins-Christie Laboratories Animal Facility. Animal studies were approved by the McGill University Animal Care Committee and performed following the Canadian Council on Animal Care guidelines.

### Sensitization & airway challenge

Mice were sensitized intraperitoneally (IP) with 100 μg OVA (Sigma-Aldrich, St. Louis, Missouri, USA) in sterile PBS with either a 10% solution of Imject Alum (Thermo Scientific, Rockford, Illinois, USA), a 50% emulsion of Complete Freund’s Adjuvant (Sigma-Aldrich) or without adjuvant on days 0 & 7. Each OVA group had a respective control group injected without OVA. All mice were lightly anaesthetized with isoflurane and challenged intranasally (IN) with 30 μl of PBS containing 50 μg of OVA on days 28, 29 & 30 and sacrificed 24 hours after the last airway challenge.

### γδ TCR antibody treatment

Balb/c mice were OVA/CFA sensitized and OVA challenged according to the protocol above, with the following exceptions: mice were alternatively challenged on days 42, 43 & 44 and received a total of two 80 μg IV injections of the UC7-13D5 γδ TCR antibody or Armenian hamster isotype control antibody (Biolegend, San Diego, California, US) 2 days and 6 hours before the first airway challenge. Flow cytometry analysis of BAL fluid and lung cells confirmed an increase in the frequency of IL-17-γδ T cells in mice receiving the γδ TCR stimulatory antibody.

### Analysis of airway hyperresponsiveness & airway inflammation

The AHR readouts, resistance and elastance, were taken from mice anaesthetized with a cocktail of xylaxine and sodium pentobarbital, followed by an injection of the paralyzing agent, pancuronium bromide. Measurements were determined using the flexiVent small animal ventilator (SCIREQ, Montreal, Quebec, Canada) by exposing mice to increasing concentrations of aerosolized methacholine. Following assessment of AHR, BAL was performed. Cells, recovered from two-1 ml PBS lavages of the airways, were pooled and red blood cells lysed before counting total cells. A portion of the cells was centrifuged onto glass slides and stained using Diff-Quick (Fisher Scientific, Ottawa, Ontario, Canada) from which a differential cell count, based on a 300 cell count from five to ten fields, was obtained. BAL fluid from the first lavage was frozen and maintained at −80°C for later cytokine/chemokine protein analysis.

### Detection of OVA-specific IgE

Preceding removal of the lungs, the chest cavity was opened and blood collected by cardiac puncture. The blood was left at room temperature for 30 min to facilitate coagulation before centrifugation and separation of serum for storage at −80°C for future analysis. OVA-specific IgE was quantified using a modified protocol of the ELISA MAX mouse IgE kit (Biolegend, San Diego, California, USA). The kit protocol was followed with the following exceptions: wells used to measure OVA-specific IgE from serum samples were coated with 100 μl of a 20 μg/ml OVA solution overnight at 4°C in place of the coating antibody. Serum samples were incubated with an equal volume of protein G sepharose overnight at 4°C. Following centrifugation, the supernatants were added to wells during the sample incubation step.

### Preparation of lung cells

The right lung was stored in RNAlater (Ambion, Carlsbad, California, USA) at −20°C for analysis by real-time PCR. A single cell suspension of lung cells was obtained from the left lung of each mouse by first mincing the lung and then incubating the tissue in 1 ml of serum-free DMEM (Life Technologies, Carlsbad, California) containing Liberase TM (100 μg/ml; Roche, Indianapolis, Indiana, USA) combined with collagenase XI (250 μg/ml), hyaluronidase 1a (1 mg/ml) and DNase I (200 μg/ml, Sigma-Aldrich) for 1 h at 37°C. The reaction was stopped with a 20 mM final concentration of EDTA [29]. Red blood cells were lysed following enzymatic digestion.

### Flow cytometric analysis

Single cell preparations of BAL and lung cells were incubated for 4 h in 1 ml of RPMI media containing 1% penicillin/streptomycin, 10% heat-inactivated fetal bovine serum (Life Technologies); 0.67 μl/ml GolgiStop from the Cytofix/Cytoperm Fixation/Permeabilization Kit (BD Biosciences, San Diego, California, USA); 50 ng/ml phorbol 12-myristate 13-acetate (PMA) and 500 ng/ml ionomycin (Sigma-Aldrich). Surface and intracellular cytokine staining were performed according to the kit protocol. Cells were double or triple stained with a combination of the following antibodies: α-CD4-Pacific Blue, α-γδ TCR-Fluorescein Isothiocyanate (GL3 clone), α-IL-17-AlexaFluor 647, α-CD3-Pacific Blue (BD Biosciences) or α-Vγ4-Fluorescein Isothiocyanate (UC3-10A6 clone) (Biolegend). Stained cells were processed using an LSRII flow cytometer (BD Biosciences) and analyzed using FlowJo software (Tree Star, Inc., Ashland, Oregon, USA).

### RNA purification & real time PCR analysis

Lung tissue was homogenized and total RNA isolated following the PureLink RNA Mini Kit (Ambion) protocol. RNA was reverse-transcribed into cDNA using Oligo dT primers and SuperScript II (Life Technologies). Real-time quantitative PCR amplification was performed with Power SYBR Green (Applied Biosystems, Warrington, UK) using the Applied Biosystems PCR system. Cycle threshold values were first normalized to *Gapdh* gene expression before quantification by the comparative threshold cycle method to obtain the gene expression levels from lungs of OVA sensitized and challenged mouse groups, relative to the saline control group [30].

### Quantitative analysis of BAL fluid mediators

BAL fluid cytokine and chemokine levels were quantified with the Q-View Imager using the 16-plex mouse cytokine screen (Quansys Biosciences, Logan, Utah, USA). IL-13 levels in the BAL fluid were quantified using the ELISA Ready-SET-Go kit (ebioscience, San Diego, California, USA).

### Statistical analysis

Data are expressed as the mean +SEM. Multiple comparisons (i.e. antigen- and adjuvant-dependent effects) were analyzed by two-way ANOVA, followed by the Holm-Sidak post hoc test. Single comparisons (between the 3 OVA-sensitized groups) were analyzed by one-way ANOVA, followed by the Holm-Sidak post hoc test. Single comparisons (between the 2 antibody treated groups) were analyzed by an unpaired, two-tailed t-test. p-values less than 0.05 were considered statistically significant. Figures and statistics were analyzed using GraphPad Prism 6 (La Jolla, California, USA).

## Results

### Enhanced airway inflammation, but lack of AHR, in mice sensitized to OVA in the presence of CFA

**Figure 1:**
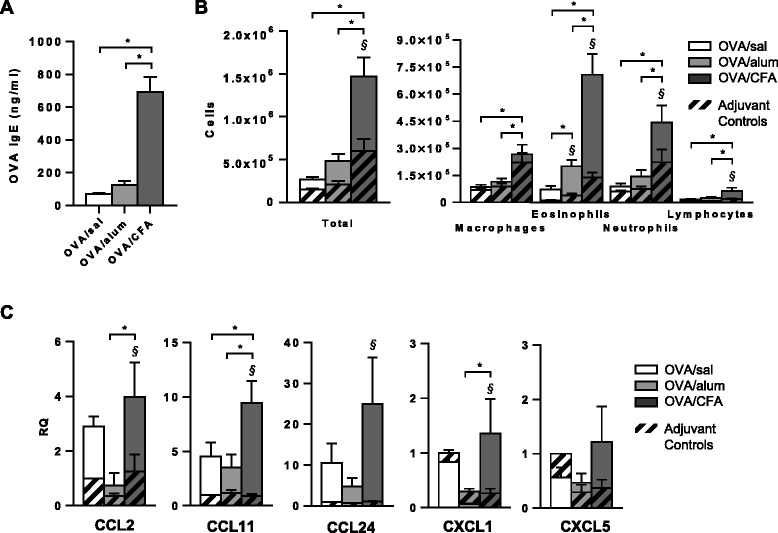
**Serum OVA-specific IgE and airway inflammatory responses are enhanced in OVA/CFA sensitized mice.** BALB/c mice were IP OVA sensitized without adjuvant (OVA/sal), or in the presence of alum (OVA/alum) or CFA (OVA/CFA). Corresponding control groups were injected with saline, alum or CFA. **(A)** Serum OVA-IgE levels. OVA-IgE was undetectable in control mice. Mean (+SEM) from 8–12 mice per group from a minimum of 3 independent experiments. One-way ANOVA, Holm-Sidak. **(B)** BAL fluid differential cell counts from OVA sensitized groups (solid bars) and their respective adjuvant controls (striped bars). Mean total cells, macrophages, eosinophils, neutrophils and lymphocytes (+SEM) are shown for 11–16 total mice from at least 3 independent experiments. Two-way ANOVA, Holm-Sidak. **(C)** Relative expression of CCL2, CCL11, CCL24, CXCL1 and CXCL5 in the lung following OVA challenge assessed by real-time PCR. Expression is presented as fold increase relative to the saline control. Results represent the means (+SEM) from 5–6 total mice per group from a minimum of 2 independent experiments. Two-way ANOVA, Holm-Sidak. **(A-C)** ^§^p < 0.05 OVA sensitized groups vs. respective controls (antigen-dependent effects), *p < 0.05 comparisons between OVA groups (adjuvant-dependent effects).

In order to establish a mixed model of allergic asthma in which the IL-17 response could be assessed within an *in vivo* Th2 environment, we intraperitoneally (IP) sensitized mice with OVA in the absence (OVA/sal group) or presence of the adjuvants, alum (OVA/alum group) or CFA (OVA/CFA group). We confirmed induction of several classic features associated with allergic airways disease and differentiated OVA-specific (§) from adjuvant-specific (*) effects (Figure 1). OVA-IgE was selectively detected in all OVA sensitized and challenged mice and was present at significantly higher levels in OVA/CFA mice (Figure 1A). Total cells, eosinophils, neutrophils and lymphocytes were significantly increased in the BAL fluid of OVA/CFA sensitized mice (solid bar) compared to the CFA control (striped bar). In contrast, inflammation was not significantly changed in OVA/sal mice and only eosinophils were significantly increased in OVA/alum sensitized mice (Figure 1B). Moreover, following OVA challenge, OVA/CFA mice had significantly more macrophages, eosinophils, neutrophils and lymphocytes, resulting in 3 and 5.5 fold more total cells recovered compared to OVA/alum and OVA/sal mice, respectively. With regard to BAL fluid cell frequencies, eosinophils were increased in all OVA-sensitized mice compared to their respective controls, primarily at the expense of macrophages (Additional file 1: Figure S1). OVA/alum sensitized and challenged mice had greater frequencies of BAL fluid eosinophils than OVA/sal mice, while OVA/CFA mice had greater frequencies of eosinophils, as well as lower frequencies of both macrophages and lymphocytes compared to OVA/sal. Regardless of adjuvant, a mixed eosinophilic/neutrophilic inflammatory profile was observed in all OVA sensitized groups following OVA challenge.

To assess possible mechanisms by which inflammatory cell recruitment differed, differences in lung chemokine expression were assessed following OVA challenge. Levels of mRNA encoding the macrophage chemoattractant, CCL2 (MCP-1), the eosinophil chemoattractants CCL11 (eotaxin-1) and CCL24 (eotaxin-2), and the neutrophil chemoattractant, CXCL1, were increased in OVA/CFA sensitized mice compared to OVA/sal and/or OVA/alum sensitized mice with these differences reaching statistical significance for CCL2, CCL11, and CXCL1 (Figure 1C). Consistent with the overall increase in BAL fluid inflammatory cell influx, mRNA levels for several chemokines were greater in OVA/CFA mice compared to OVA/sal and/or OVA/alum mice. Moreover, OVA/CFA mice were also the only group to show any significant OVA-specific increases in chemokine expression, with the exception of the neutrophil chemoattractant, CXCL5.

**Figure 2:**
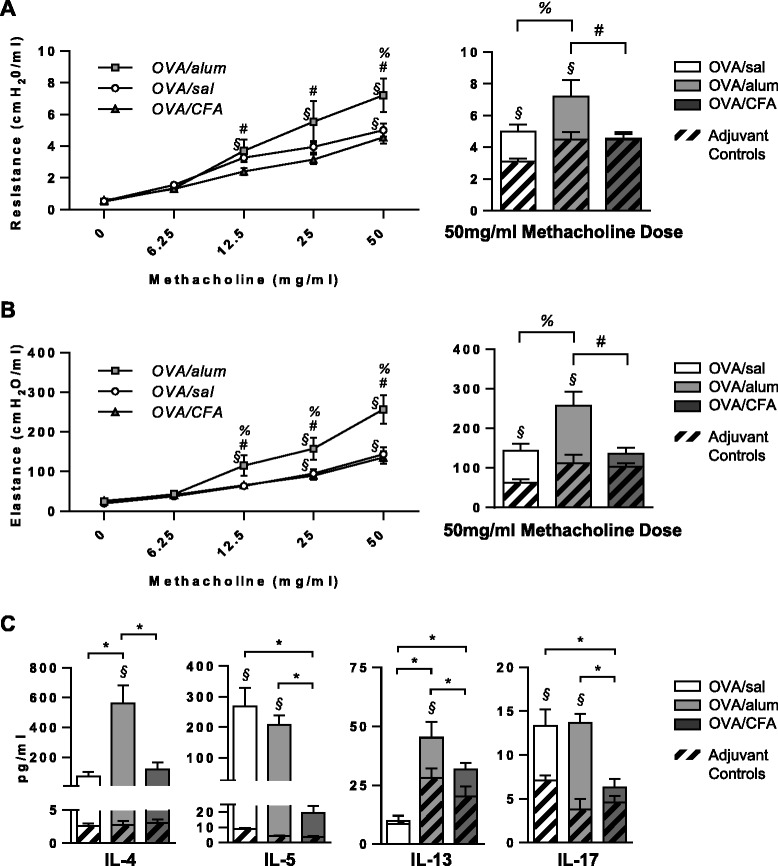
**AHR is absent in OVA/CFA sensitized mice and Th1/Th2 BAL fluid cytokine levels are influenced by the adjuvant used during OVA sensitization.** Total lung resistance and elastance were assessed 24 h after the last airway OVA challenge. Mean (±SEM) respiratory system **(A)** resistance and **(B)** elastance to increasing concentrations of methacholine (left panels) for the 3 OVA-sensitized mouse groups, as well as at the 50 mg/ml concentration (right panels) for all 6 groups. Control groups were removed from left panel figures for clarity. Data from 8–14 total mice per group from 2 independent experiments. **(C)** Mean BAL fluid levels (+SEM) of IL-4, IL-5, IL-13 & IL-17 were assessed from 7–12 total mice per group, from at least 2 independent experiments. **(A-C)** Two-way ANOVA, Holm-Sidak. ^§^p < 0.05 OVA sensitized groups versus respective controls (antigen-dependent effects); *p < 0.05 comparisons within the OVA sensitized groups (adjuvant-dependent effects), specifically ^#^p < 0.05 OVA/alum vs. OVA/CFA, ^%^p < 0.05 OVA/alum vs. OVA/sal.

Following OVA challenge, airway resistance (Figure 2A) and elastance (Figure 2B) were significantly increased in OVA/alum sensitized mice compared to OVA/CFA mice, across multiple concentrations of methacholine (left panels), and to OVA/sal mice at the highest dose (right panels). Moreover, the OVA-dependent increases (§) in airway resistance and elastance that were observed in OVA/alum (and OVA/sal) sensitized mice were completely absent in OVA/CFA sensitized mice. Thus, although OVA-IgE and airway inflammation were significantly enhanced in OVA/CFA sensitized mice (Figure 1A, B), AHR was absent. Although adjuvant-only controls showed subtle differences in respiratory resistance and elastance at the 50 mg/ml methacholine dose (Figure 2A and B, right panels), these differences were not statistically different. BAL fluid was analyzed for Th1, Th2 and Th17 related cytokines. Levels of IFN-γ were generally low in all groups (data not shown). In OVA/sal sensitized mice, OVA challenge increased levels of IL-5 and IL-17 (Figure 2C). OVA/alum sensitized mice had increased IL-4, IL-5, IL-13 and IL-17 levels compared to alum-sensitized mice, post airway challenge. OVA/alum mice also had significantly greater levels of these cytokines compared OVA/sal and/or OVA/CFA mice. Interestingly, OVA/CFA sensitized mice showed no differences in cytokine levels compared to CFA-sensitized mice.

### When emulsified with OVA at sensitization, CFA increases IL-17-γδ T cell numbers in the BAL fluid

**Figure 3:**
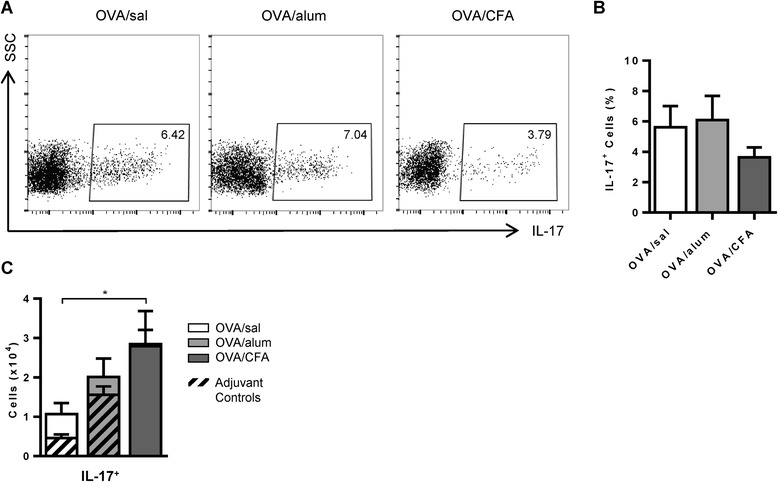
**IL-17 expressing cells are present in the BAL fluid of all OVA sensitized and challenged mice.** Cells were stimulated with PMA/ionomycin and stained to detect IL-17. **(A)** Representative flow cytometry plots are shown. **(B)** The frequencies of IL-17 expressing cells for the OVA groups are shown. One-way ANOVA, Holm-Sidak. **(C)** The total number of IL-17 expressing cells are shown for OVA groups. Two-way ANOVA, Holm-Sidak. Mean values (+SEM) from 7–11 total mice per group, from 2 independent experiments. **(B-C)** *p < 0.05 comparisons between OVA groups.

Following OVA challenge, IL-17 expressing cells were present in the BAL fluid of all mice, regardless of the conditions of OVA sensitization (Figure 3A). Although there was a trend toward a reduced frequency of IL-17^+^ cells in mice sensitized with OVA/CFA, this difference did not reach statistical significance (Figure 3B). Nevertheless, when taking into account the overall inflammatory cell influx into the BAL fluid, OVA/CFA sensitized mice had larger numbers of IL-17^+^ cells compared to the other OVA sensitized groups, though this difference was only significant when compared to the OVA/sal group (Figure 3C). No antigen-dependent differences were observed in the OVA sensitized mouse groups compared to their respective controls.

**Figure 4:**
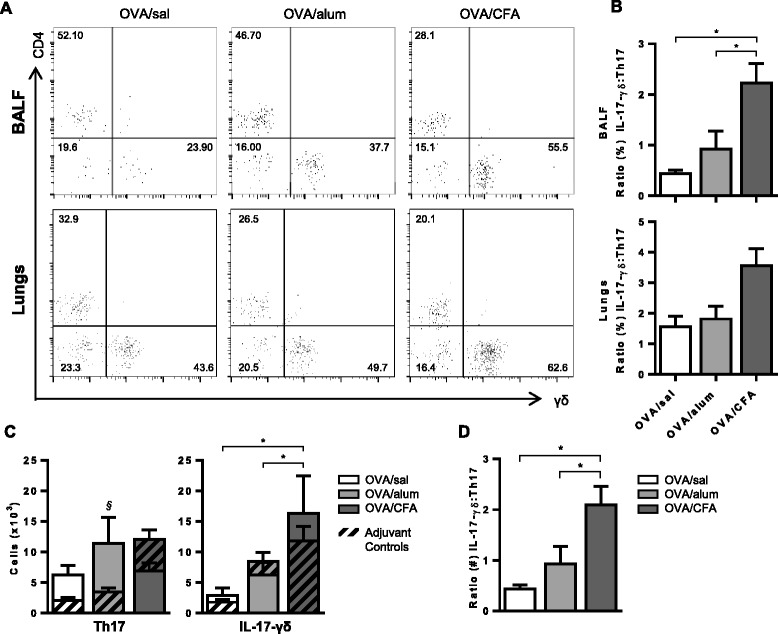
**OVA/CFA sensitized mice have more γδ** ^**+**^ **IL-17** ^**+**^ **T cells in the BAL fluid.** BAL fluid cells were stimulated with PMA/ionomycin and triple stained with α-CD4, α-γδ TCR and α-IL-17 antibodies. **(A)** Representative flow cytometry plots of cells in BAL fluid (top panel) and lung (bottom panel) gated first to identify IL-17^+^ cells and subsequently to identify frequencies of CD4 and γδ T cells. **(B)** The frequency distribution of IL-17^+^ cells within the CD4 and γδ T cell populations is presented as the ratio (γδ/CD4) of these cells. One-way ANOVA, Holm-Sidak. **(C)** Total numbers of CD4^+^IL-17^+^ and γδ^+^IL-17^+^ populations calculated from the frequency of these cells and the total cell counts are shown. Two-way ANOVA, Holm-Sidak. **(D)** The distribution of total γδ^+^IL-17^+^ and CD4^+^IL-17^+^ T cells is presented as the ratio (γδ/CD4) of these cells. One-way ANOVA, Holm Sidak. **(B-D)** Data are from 7–11 total mice per group from at least 2 independent experiments. *p < 0.05 comparisons between OVA groups (adjuvant-dependent effects), ^§^p < 0.05 OVA sensitized groups versus respective controls (antigen-dependent effects).

Several cell types, including Th17 cells [24,31], γδ T cells [27,28] and alveolar macrophages [32] have been identified as sources of IL-17 in murine models of allergic airways disease. Following OVA challenge, we identified the major IL-17 producing cell(s) in both the BAL and lungs of OVA/sal, OVA/alum and OVA/CFA sensitized mice. After gating on IL-17^+^ cells, the major sources of IL-17 were identified as CD4^+^ (Th17) and γδ (IL-17-γδ) T cells, though the ratio differed depending on the conditions of OVA sensitization (Figure 4A). The ratio of the frequency of IL-17-γδ to Th17 cells in the BAL fluid established that Th17 cells were the major source of IL-17 in OVA/sal sensitized mice; that Th17 and IL-17-γδ T cells contributed almost equally to IL-17 production in OVA/alum sensitized mice; and that IL-17-γδ T cells were the main producers of IL-17 in OVA/CFA sensitized mice (Figure 4B). Similar total numbers of Th17 cells were present in the BAL fluid of all OVA sensitized and challenged mice (Figure 4C), but greater numbers of total IL-17-γδ T cells were recovered from OVA/CFA sensitized mice compared to OVA/alum and OVA/sal mice. Only the OVA/alum group had significantly greater numbers of Th17 cells compared to its respective control (Figure 4C, left panel), whereas antigen-dependent differences were absent for total IL-17-γδ T cells (Figure 4C, right panel). The ratios of total IL-17-γδ to Th17 cells for all OVA sensitized mouse groups were similar to the ratios of the frequencies of the same cells (Figure 4D), with OVA/CFA sensitized mice having a significantly larger ratio to both OVA/sal and OVA/alum mice. The median fluorescence intensity (MFI) of IL-17 was greater in IL-17-γδ T cells compared to Th17 cells from the BAL fluid (Additional file 1: Figure S2A, B) for all OVA sensitized groups, indicating that, while the frequencies of cell types expressing IL-17 were modified by the type of adjuvant present at sensitization, the amount of IL-17 expressed by these individual cells was not affected.

### Activating IL-17-γδ T cells leads to inhibition of airway eosinophilia and AHR

**Figure 5:**
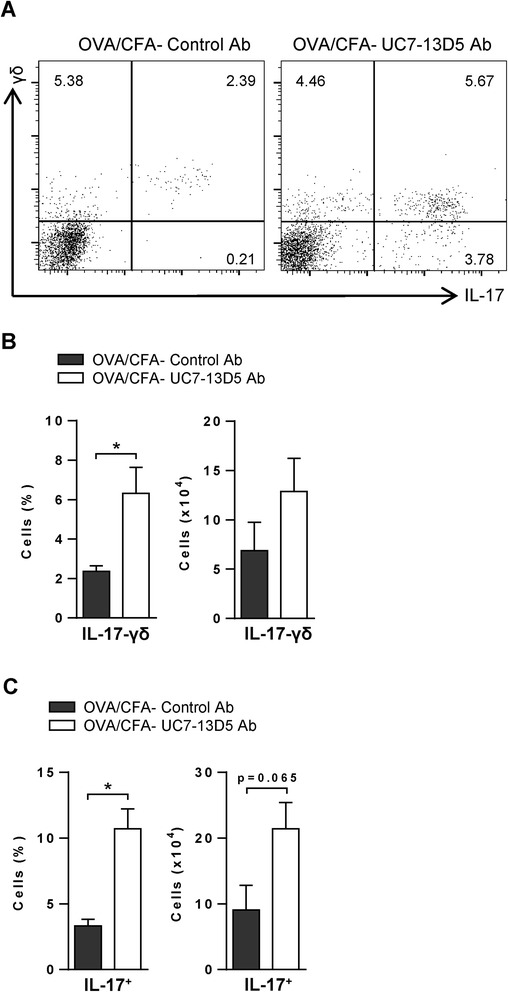
**Frequencies of total IL-17 expressing cells and of IL-17-γδ cells are increased in OVA/CFA sensitized mice treated with a γδ TCR stimulatory antibody.** OVA/CFA sensitized mice were IV injected with a γδ TCR stimulatory antibody (UC7-13D5) or isotype control before airway challenge. BAL fluid cells were stimulated with PMA/ionomycin and stained with α-γδ TCR and α-IL-17 antibodies. **(A)** Representative flow cytometry plots of BAL fluid cells. The mean frequencies (left panels) and numbers (right panels) of **(B)** IL-17-γδ and **(C)** IL-17^+^ BAL fluid cells are presented for 5 total mice per group from 2 independent experiments. **(B-C)** Unpaired, two-tailed t-test. *p < 0.05.

**Figure 6:**
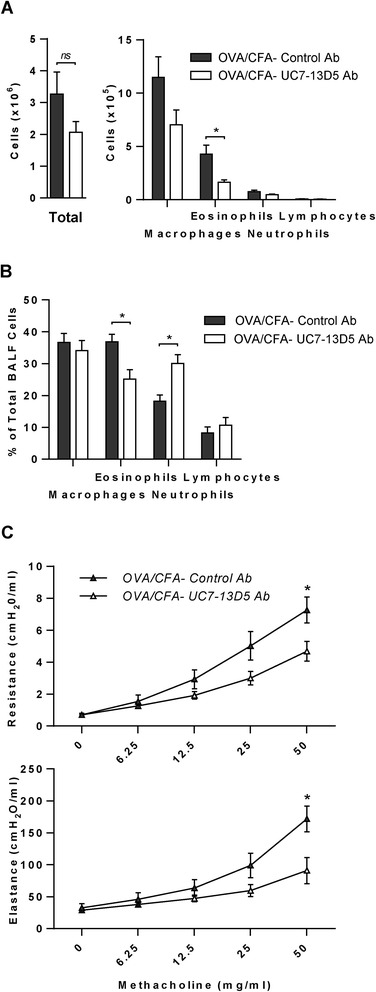
**OVA/CFA sensitized mice receiving a γδ TCR stimulatory antibody, have reduced airway eosinophilia and AHR.** OVA/CFA sensitized mice were IV injected with a γδ TCR (UC7-13D5) stimulatory antibody or isotype control before airway challenge. The mean **(A)** total cell counts and **(B)** frequencies of macrophages, eosinophils, neutrophils and lymphocytes (+SEM) are shown for 5 total mice per group from 2 independent experiments. **(C)** Total lung resistance and elastance were assessed 24 h after the last airway OVA challenge. Mean (±SEM) respiratory system resistance and elastance to increasing concentrations of methacholine are shown. **(A-C)** Unpaired, two-tailed t-test. *p < 0.05, ns = not significant.

To examine the impact of elevated numbers of IL-17-γδ T cells present in mice sensitized with OVA/CFA, these mice were treated with a γδ TCR antibody, UC7-13D5. Consistent with a study demonstrating that this antibody activates γδ T cells *in vitro* and *in vivo* [33], UC7-13D5 treated OVA/CFA sensitized mice exhibited increased frequencies of BAL fluid IL-17-γδ T cells (Figure 5A, B left panel) and, interestingly, IL-17^+^ cells (Figure 5A, C left panel), post airway challenge. There was also a positive trend for the number of IL-17-γδ T cells and IL-17^+^ T cells to be increased in UC7-13D5-treated mice (right panels of Figure 5B and C). The majority of the IL-17-γδ T cells in the UC7-13D5-treated mice were of the Vγ4 subset (Additional file 1: Figure S3A, B), which has previously been shown to inhibit AHR and airway inflammation in murine models of asthma [28]. In agreement, BAL fluid eosinophils (Figure 6A, B) were significantly decreased in antibody treated mice compared to recipients of isotype control antibodies. Moreover, the frequency of BAL fluid neutrophils was also increased (Figure 6B), which may reflect the increase in total IL-17 expressing cells in mice treated with the UC7-13D5 antibody. Finally, both respiratory resistance and elastance were diminished in OVA/CFA mice treated with the UC7-13D5 antibody, again consistent with a negative regulatory role for IL-17-γδ T cells (Figure 6C).

## Discussion

In rodent models of allergic airways disease, the types of antigen and adjuvant, as well as their concentration, route and timing of delivery can impact the nature of the ensuing immune response. Historically, the most widely used murine models of allergic asthma and rhinitis have relied upon IP injections of OVA adsorbed to an aluminum-based adjuvant [34,35]. This class of adjuvant has been used extensively in animal models of allergic airways disease for its ability to induce a Th2-biased immune response [20,36]. In contrast, CFA has been recognized as a Th17-promoting adjuvant and is comprised of a light mineral oil, a surfactant agent, and heat-killed *Mycobacterium tuberculosis* (*Mtb*) that creates an emulsion when mixed with an aqueous solution [37]. A great deal of our understanding of the IL-17/CFA relationship has come from experimental autoimmune encephalomyelitis models of multiple sclerosis [38]. Nevertheless, antigen sensitization with CFA has rarely been used in models of allergic airways disease. Our goal was to assess how the choice of adjuvant—alum or CFA—affected Th2 and Th17 type inflammatory responses in the airways post antigen challenge.

CFA has been widely used in experimental studies for its unsurpassed ability to enhance antibody production against a number of different antigens [37]. This was confirmed in our analysis as OVA/CFA sensitized mice, post airway challenge, had significantly greater levels of serum OVA IgE compared to both OVA/sal and OVA/alum sensitized mice. Abundant data demonstrate that, independent of the mouse strain, mice IP sensitized with OVA/alum have robust airway eosinophilia post challenge [34,35]. Nevertheless, the airways of C57Bl/6 mice sensitized IP with OVA/alum or OVA/CFA can also exhibit a mixed eosinophilic/neutrophilic inflammatory profile of similar magnitude following acute antigen challenge [22]. Our data indicate that Balb/c mice IP sensitized to OVA with alum or CFA yield mixed eosinophilic/neutrophilic inflammatory profiles where equal *ratios* of both cell types were recovered in the BAL fluid. These data suggest that both Th2 and IL-17 responses, which are associated with airway eosinophilia [39] and neutrophilia [21], respectively, may be induced with either adjuvant. Although AHR is often correlated with airway inflammation, there is evidence from humans [40,41], as well as murine models [42,43], that AHR can occur independently of inflammation. Our data indicate that mice sensitized with OVA/CFA had significantly more inflammation following OVA challenge, as assessed from the BAL fluid. Nevertheless, both antigen- and adjuvant-specific increases in airways resistance and elastance were completely absent in these mice. Comparatively, OVA/alum sensitized mice exhibiting moderate inflammation, had both antigen- and adjuvant-dependent increases in AHR. These differences in airways resistance and elastance may be related to BAL fluid levels of IL-4 and IL-13, which were elevated in only the OVA/alum sensitized mice. Intratracheal instillations of IL-13 to naïve mice [44] or IL-4 to IL-13 deficient mice can induce AHR [21]. Interestingly, the same studies showed IL-4 and IL-13 to be potent inducers of airway eosinophilia, whereas in our study, airway hyporesponsive, highly eosinophilic OVA/CFA sensitized mice had comparatively lower BAL fluid levels of both cytokines. We therefore considered other mediators to provide a possible explanation for the disconnect between AHR and inflammation observed in OVA/CFA mice. The ability of IL-17 to act as both a positive and negative regulator of asthma [27], as well as the ability of CFA to induce IL-17 in models of experimental autoimmune encephalomyelitis [45] made this particular cytokine a strong candidate. While IL-17 alone may not induce AHR in mice, under some circumstances it can promote Th2-dependent AHR and inflammation [26].

We identified the cellular source(s) of IL-17 in mice sensitized under different adjuvant conditions. Following OVA challenge, IL-17 was expressed predominantly by a combination of CD4^+^ and γδ T cells in the BAL fluid and lungs (Figure 4A) of all OVA-sensitized and challenged mice. However, in OVA/sal sensitized mice, IL-17 was produced primarily by CD4^+^ T cells. Alum or CFA, given at the time of sensitization, affected this distribution by skewing IL-17 expression toward γδ T cells. *Mtb*, an essential component of CFA, preferentially induces IL-17 expression from γδ T cells rather than CD4^+^ T cells through activation of the Nalp3 inflammasome [45,46]. Moderate skewing of IL-17 production toward γδ T cells also occurred in mice sensitized with OVA/alum. This may reflect the ability of alum to activate the Nalp3 inflammasome [35,47], albeit less potently than CFA, or depend on the quantity of alum used at sensitization.

Our data demonstrating that IL-17-γδ T cells, Th17 cells (Figure 4C) and total IL-17^+^ cells (Figure 3C) recovered in the BAL fluid did not necessarily coincide with BAL fluid levels of IL-17 (Figure 2C), are consistent with data from other groups [24,28]. For example, IL-17 levels in the BAL fluid of both airway and IP sensitized mice were undetectable at a time point when total Th17 cells, assessed by flow cytometry, were significantly higher in airway sensitized mice [24]. Thus, apparent differences in IL-17 cytokine levels relative to IL-17 expressing cells may simply reflect the timing of assessment, as the ability to detect IL-17 in the BAL fluid may be quite transient [24,28], whereas detection of cells with the potential to produce IL-17 may be more stable/longlasting.

In murine models of allergic asthma, IL-17 expression is most often associated with CD4^+^ T cells, although γδ T cells and macrophages can also express IL-17 [24,28,32]. Several factors, including the quantity of cytokine produced, as well as the timing and location and cell source, likely impact the outcome of IL-17 production. For example, while Th17 cells appear to promote Th2 responses, such as eosinophil recruitment to the airways, we and others have shown that IL-17-γδ T cells actually inhibit Th2-associated AHR and/or inflammation [27]. Murdoch et al. specifically showed that 64% of IL-17-γδ T cells were of the Vγ4^+^ subset [28]. This specific subset of γδ T cells actively suppresses AHR [48] and may accomplish this without affecting inflammation [49]. We similarly showed a population of IL-17^+^Vγ4^+^ cells constituting a large majority of the IL-17-γδ T cells in the BAL fluid of OVA/CFA sensitized mice (Additional file 1: Figure S3). Thus, the prominent IL-17-γδ T cell population recovered in OVA/CFA sensitized mice may explain, at least in part, the absence of AHR, despite the robust inflammatory response. For these same reasons, regulatory T cells are not likely to be involved in the suppression of AHR, due to their well-established role in suppressing inflammation [50].

In order to better understand how IL-17-γδ T cells regulated airway inflammatory responses in OVA/CFA sensitized mice, we treated these mice with a γδ TCR stimulatory antibody (UC7-13D5), the outcome of which was a substantial increase in the frequency of IL-17-γδ T cells accompanied by reductions in both AHR and eosinophil influx. We were not surprised to find that the increase in IL-17-γδ T cells corresponded to a decrease in AHR, in particular since the majority of the IL-17-γδ T cells induced in OVA/CFA sensitized mice appeared to be the Vγ4, inhibitory subset. Unlike the inhibition of AHR, the reduced number of airway eosinophils in UC7-13D5 treated mice was unexpected as OVA/CFA sensitized mice that followed the 4 week sensitization and challenge protocol had increased IL-17-γδ T cells in the BAL fluid (Figure 4C) and suppressed AHR (Figure 2A, B), but had highly inflamed airways, including abundant eosinophils (Figure 1B). γδ T cells (IL-17^+^ and Vγ4^+^) are described as having either an inhibitory or no effect on airway eosinophilia, based on different OVA models of allergic airways disease [28,48,49]. Careful examination of the literature indicates that this may be a temporal issue. In these OVA models, γδ T cells are consistently elevated in numbers at the peak of eosinophil recruitment (24–48 hours after the last airway challenge), suggesting that IL-17-γδ T cells do not have an immediate inhibitory effect on the influx of eosinophils. However, in the days following antigen challenge, (i.e. recovery phase), IL-17-γδ T cells have specifically been shown to decrease airway eosinophil numbers and further attenuate AHR [28]. Where γδ T cells are reported as having no effect on eosinophil recruitment, measurements were taken at the peak of inflammation and compared to mice that received a Vγ4 neutralizing antibody [49], which would neutralize a population of cells that has yet to influence airway eosinophil numbers. In contrast, in our studies, the UC7-13D5 antibody, that was given pre-challenge to OVA/CFA sensitized mice, stimulated and thus activated the γδ T cells, which may have allowed them to reduce airway eosinophil numbers even at 24 hours after the last airway challenge. Further studies are required to confirm these findings. IL-17-γδ T cells have been reported to increase IL-17 production from Th17 and other cell types [38,51,52]. This may explain the increase in total IL-17^+^ cells recovered in the BAL fluid of mice treated with the UC7-13D5 antibody. Consistent with the literature, the frequencies of neutrophils in UC7-13D5 treated mice (Figure 6B) reflected the increase in IL-17^+^ cells (Figure 5C) [24,53]. However, the overall neutrophil cell count remained low and did not differ between UC7-13D5 and isotype control antibody treated OVA/CFA sensitized mice (Figure 6A).

The UC7-13D5 γδ TCR antibody has previously been used to functionally deplete γδ T cells in vivo [54]. Though we used comparable concentrations of this antibody, as discussed above, our data indicate that the target cells were neither neutralized, nor functionally depleted. In fact, our data are consistent with data provided by Koenecke, et al. demonstrating that γδ T cells are activated by UC7-13D5 [33]. To study γδ T cells in OVA/CFA sensitized mice, two different antibodies were used: mice were injected with the UC7-13D5 γδ TCR antibody, followed by γδ T cell detection by flow cytometry using the GL3 γδ TCR antibody. While the treatment and staining antibodies differ, our data and others [33] suggest that the GL3 and UC7-13D5 antibodies compete for the same γδ TCR epitope, as the γδ MFI was lower, but not to the extent of depletion, in BAL fluid cells recovered from UC7-13D5 antibody compared to control antibody treated mice (Figure 5). This was also the case for the Vγ4 staining antibody (UC3-10A6 clone), but appeared to compete to a lesser degree with the UC7-13D5 antibody (Additional file 1: Figure S3). In either case, it is clear that the γδ T cells were not depleted. Furthermore, as discussed above, there was a significant increase in the expression of IL-17 within the γδ T cells from UC7-13D5 treated mice, while the frequency of total γδ T cells was unchanged compared to that in mice treated with the isotype control (data not shown).

Although the adjuvants, alum and CFA, significantly impacted the distribution of IL-17 expression between the CD4^+^ and γδ T cell populations, the median fluorescence intensity (MFI) of IL-17 within each cell type was unaffected. In fact, the MFI of IL-17 in BAL fluid γδ T cells was significantly greater than in CD4^+^ T cells for all three OVA sensitized groups. Lockhart et al. discussed the frequency of IL-17 expression within the same cell populations in a murine model of *Mtb* infection [46]. Although they did not directly address the MFI of IL-17, it too appeared to be higher in γδ T cells than in CD4^+^ T cells. Therefore, the level of expression of IL-17 in this model, as well as in our model of allergic airways disease, may be applicable to other physiological or pathological lung conditions. We previously demonstrated that a low dose of IL-17 augments IL-13-induced airway inflammatory responses, while a higher dose of IL-17 attenuates these responses [27]. In parallel, the “low dose” of IL-17 produced by CD4^+^ T cells in the current study may have augmented disease while our data suggest that the “high dose” of IL-17 produced by γδ T cells attenuated disease.

## Conclusions

Altogether, our data demonstrate that intraperitoneal OVA sensitization with and without the adjuvants, alum and CFA, regulated the profile of IL-17 producing cells localized to the lung post OVA challenge, providing insight into potential mechanisms by which IL-17 may negatively regulate allergic airways disease. Notwithstanding the large influx of inflammatory cells to the airways, AHR was completely absent in OVA/CFA sensitized mice post OVA challenge. This lack of AHR coincided with an increase in IL-17-γδ T cells in the BAL fluid, in line with previous data demonstrating that these cells are potent inhibitors of airway inflammatory responses [27,28]. While the dual role of IL-17 makes it a complex target for drug development, our data suggest that treatments specifically focused on enhancing the IL-17-γδ T cell population may be beneficial for asthmatics.

## Additional file

Additional file 1: Figure S1. Frequencies of macrophages, eosinophils, neutrophils and lymphocytes in the BAL fluid of OVA sensitized mice and their respective controls. 11–16 mice from at least 3 independent experiments. Two-way ANOVA, Holm-Sidak. ^§^p < 0.05 OVA groups vs. their respective controls; *p < 0.05 versus OVA/sal, ^p < 0.05 versus CFA. Additional relevant comparisons were not significantly different. **Figure S2.** Frequencies of IL-17^+^ cells within the γδ T cell population and the MFI of IL-17 are greater than those of CD4^+^ T cells from the BAL fluid. Cells were triple stained with α-CD4, α-γδ TCR and α-IL-17 antibodies. (A) Representative flow cytometry plots of BAL fluid cells from OVA sensitized groups. (∆) indicates the MFI of IL-17 within the IL-17-expressing CD4^+^ and γδ TCR^+^ cell populations on a log scale. (B) The MFI of IL-17 expression within the IL-17 expressing CD4^+^ and γδ T cell populations are shown. Flow cytometry plots and mean values (+SEM) are from 7–9 mice per group from at least 2 independent experiments. Two-way ANOVA, Holm-Sidak. *p < 0.05. **Figure S3.** The majority of IL-17-γδ T cells in the BAL fluid of OVA/CFA sensitized mice are of the Vγ4 subset and increase in frequency with activation by the γδ TCR antibody. OVA/CFA sensitized mice were IV injected with a γδ TCR (UC7-13D5) stimulatory antibody or isotype control before airway OVA challenge. BAL fluid cells were double stained with α-IL-17 and either the α-γδ TCR or α-Vγ4 antibodies. Representative flow cytometry plots of the frequencies of γδ^+^IL-17^+^ and Vγ4^+^IL-17^+^ cells from recipients of (A) control or (B) UC7-13D5 antibody are shown within the total live BAL fluid cell population and are representative of 2–3 mice per group.

## Abbreviations

AHR: Airway hyperresponsiveness
BAL: Bronchoalveolar lavage
IP: Intraperitoneal
IN: Intranasal
IV: Intravenous
MFI: Median fluorescence intensity
*Mtb*: *Mycobacterium tuberculosis*
