# Supplementary figures and images for: Adjuvant-dependent regulation of interleukin-17 expressing γδ T cells and inhibition of Th2 responses in allergic airways disease
Source: Respir Res. 2014 Aug 14;15(1):90. doi: 10.1186/s12931-014-0090-5 (PMC4151193; doi:10.1186/s12931-014-0090-5)

SUPPLEMENTAL FIGURE 1

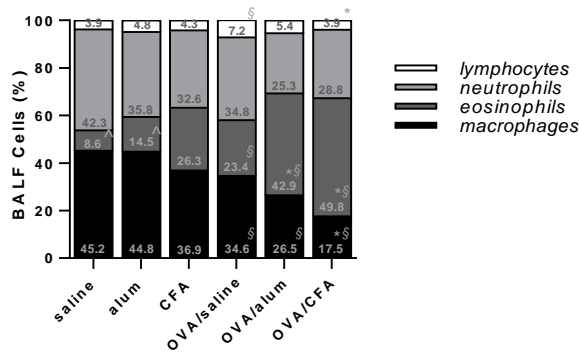

SUPPLEMENTAL FIGURE 2

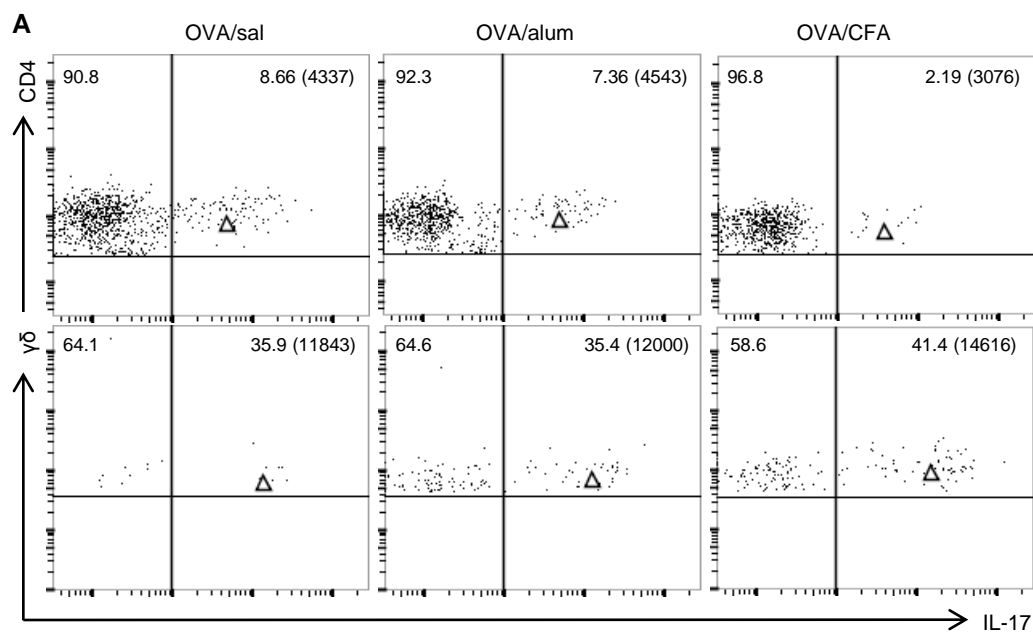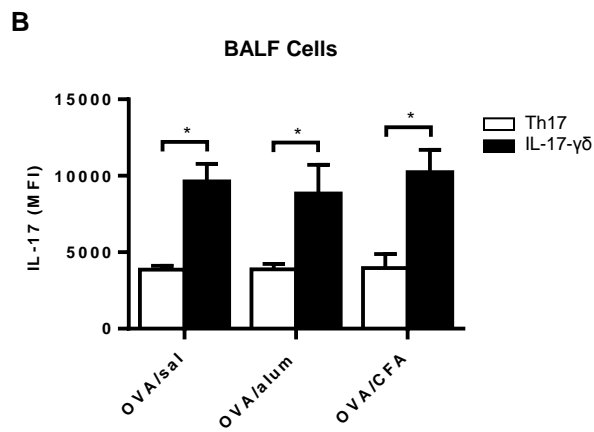

SUPPLEMENTAL FIGURE 3

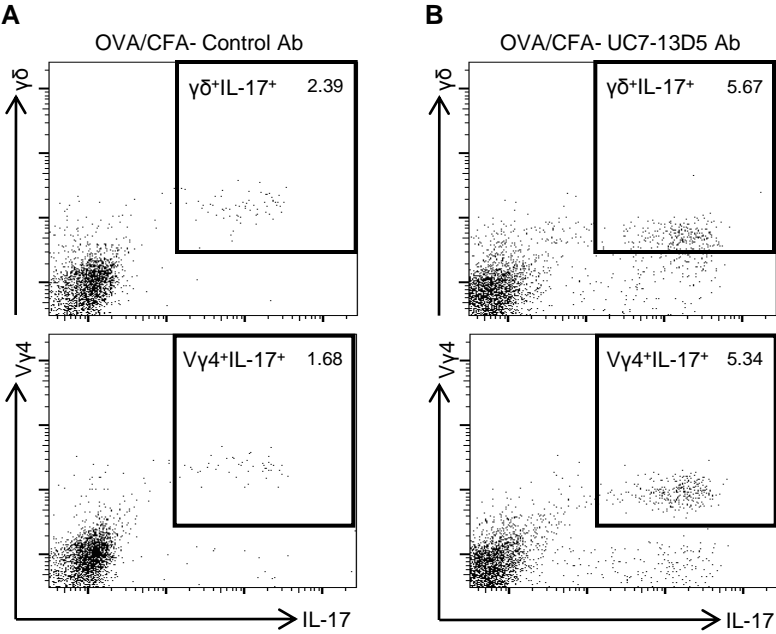

Supplement: Additional file 1: Figure S1. — Frequencies of macrophages, eosinophils, neutrophils and lymphocytes in the BAL fluid of OVA sensitized mice and their respective controls. 11–16 mice from at least 3 independent experiments. Two-way ANOVA, Holm-Sidak. §p < 0.05 OVA groups vs. their respective controls; *p < 0.05 versus OVA/sal, ^p < 0.05 versus CFA. Additional relevant comparisons were not significantly different. Figure S2. Frequencies of IL-17+ cells within the γδ T cell population and the MFI of IL-17 are greater than those of CD4+ T cells from the BAL fluid. Cells were triple stained with α-CD4, α-γδ TCR and α-IL-17 antibodies. (A) Representative flow cytometry plots of BAL fluid cells from OVA sensitized groups. (∆) indicates the MFI of IL-17 within the IL-17-expressing CD4+ and γδ TCR+ cell populations on a log scale. (B) The MFI of IL-17 expression within the IL-17 expressing CD4+ and γδ T cell populations are shown. Flow cytometry plots and mean values (+SEM) are from 7–9 mice per group from at least 2 independent experiments. Two-way ANOVA, Holm-Sidak. *p < 0.05. Figure S3. The majority of IL-17-γδ T cells in the BAL fluid of OVA/CFA sensitized mice are of the Vγ4 subset and increase in frequency with activation by the γδ TCR antibody. OVA/CFA sensitized mice were IV injected with a γδ TCR (UC7-13D5) stimulatory antibody or isotype control before airway OVA challenge. BAL fluid cells were double stained with α-IL-17 and either the α-γδ TCR or α-Vγ4 antibodies. Representative flow cytometry plots of the frequencies of γδ+IL-17+ and Vγ4+IL-17+ cells from recipients of (A) control or (B) UC7-13D5 antibody are shown within the total live BAL fluid cell population and are representative of 2–3 mice per group. [file 12931_2014_90_MOESM1_ESM.pdf]
